# Successful Use of mRNA-Nucleofection for Overexpression of Interleukin-10 in Murine Monocytes/Macrophages for Anti-inflammatory Therapy in a Murine Model of Autoimmune Myocarditis

**DOI:** 10.1161/JAHA.112.003293

**Published:** 2012-12-19

**Authors:** Oliver Zimmermann, Jörg M Homann, Anna Bangert, Anna-Maria Müller, Georgi Hristov, Stefan Goeser, Juliane M. Wiehe, Stefan Zittrich, Wolfgang Rottbauer, Jan Torzewski, Gabriele Pfitzer, Hugo A. Katus, Ziya Kaya

**Affiliations:** Cardiovascular Center Oberallgäu, Kempten, Germany (O.Z., J.T.); Department of Internal Medicine II, University of Ulm, Ulm, Germany (O.Z., J.M.H., J.M.W., W.R.); Department of Internal Medicine III, University of Heidelberg, Heidelberg, Germany (A.B., A.-M.M., G.H., S.G., H.A.K., Z.K.); Institute of Vegetative Physiology, University of Cologne, Cologne, Germany (S.Z., G.P.)

**Keywords:** cell therapy, heart failure, inflammation, interleukin-10, myocarditis

## Abstract

**Background:**

Overexpression of interleukin-10 (IL-10) in murine CD11b^+^ monocytes/macrophages via GMP-adapted mRNA-nucleofection was expected to improve clinical outcome and reduce adverse side effects in autoimmune myocarditis. This study represents the proof of principle for a novel anti-inflammatory therapy using overexpression of IL-10 in murine monocytes/macrophages by mRNA-nucleofection for the treatment of autoimmune myocarditis.

**Methods and Results:**

Autoimmune myocarditis was induced in A/J mice by subcutaneous immunization with troponin I. CD11b^+^ monocytes/macrophages were isolated from the peritoneum and IL-10 was overexpressed by mRNA-nucleofection. These cells were injected intravenously. Myocardial inflammation was assessed via histological and immunohistochemical examinations. Myocardial fibrosis was analyzed with Masson's trichrome staining. Antitroponin I antibodies were determined within the serum. Physical performance was evaluated using a running wheel and echocardiography. In vitro overexpression of IL-10 in CD11b^+^ monocytes/macrophages resulted in a 7-fold increased production of IL-10 (n=3). In vivo higher levels of IL-10 and less inflammation were detected within the myocardium of treated compared with control mice (n=4). IL-10–treated mice showed lower antitroponin I antibodies (n=10) and a better physical performance (n=10).

**Conclusions:**

Application of IL-10–overexpressing CD11b^+^ monocytes/macrophages reduced inflammation and improved physical performance in a murine model of autoimmune myocarditis. Thus, the use of genetically modified monocytes/macrophages facilitated a targeted therapy of local inflammation and may reduce systemic side effects. Because the nucleofection technique is GMP adapted, an in vivo use in humans seems basically feasible and the transfer to other inflammatory diseases seems likely.

## Introduction

Local inflammation as seen in rheumatoid arthritis or Crohn disease represents a therapeutic challenge. While immunosuppressive therapy is often based on steroidal or nonsteroidal drugs,^1,2^ in severe courses, chemotherapeutic agents, monoclonal antibodies, and stem cell therapy will be administered.^3–5^ Collateral damage is caused by systemic and unspecific action of therapeutic agents that is also directed against nearby healthy tissues.^6,7^ Because the underlying disease often requires long-term treatment, adverse effects could limit the therapy or even trigger secondary diseases. To avoid these side effects, a local and targeted anti-inflammatory therapy could represent a promising therapeutic approach.

Myocarditis is a relatively common cause of acute heart failure in the young for which an efficient and specific therapy is lacking.^8^ Although most patients recover completely, some present with a deteriorating course. Despite intensive pharmacological and device-based therapies, chronic heart failure is related with high morbidity and mortality. New therapeutics such as ventricular assist devices, cardiac resynchronization, an implantable cardioverter-defibrillator, or heart transplantation are complex and expensive.^9^ For most patients with myocarditis, efficient and selective immunomodulating therapies are still needed. Thus, nonspecific anti-inflammatory medication, drugs for heart failure, and off-label therapies are used.^8,10,11^

Interleukin-10 (IL-10 [cytokine-synthesis inhibitory factor]) is a strong anti-inflammatory cytokine that is produced by monocytes/macrophages, Th2 lymphocytes, and regulatory T cells.^12^ It mediates its effects via reduction in antigen presentation and inhibition of T-cell activation. Furthermore, IL-10 inhibits the production of proinflammatory cytokines by Th1 lymphocytes and improves survival, proliferation, and antibody production of B lymphocytes.^12–14^ In this way, IL-10 protects the organism against an uncontrolled inflammatory burst. Modulation of IL-10 expression and action in different cell types could represent a promising therapeutic approach.^15^ IL-10 has been demonstrated to play an important role in the pathogenesis of experimental myocarditis.^16^

Monocytes and macrophages are crucial participants in inflammation and autoimmune myocarditis. They represent excellent candidates for a targeted cell therapy because they selectively migrate into inflamed tissues and may release anti-inflammatory mediators locally. In a clinical setup, monocytes could easily be isolated from the patient in peripheral blood samples according to a standard protocol that does not involve relevant invasive or painful procedures. Because the cells were isolated from the patient, an application within the autologous organism becomes possible; thus, cell rejection or graft-versus-host effects would not be expected.

There are several studies that used IL-10 overexpression for therapy of inflammatory processes,^17–19^ but gene transfer is still challenging in a clinical in vivo setting. There are studies using viral vectors (eg, adenoviral, adeno-associated viral [AAV]), but these approaches are limited by their side effects, such as high viral titers not only in targeted tissue but also in nearby healthy organs (eg, liver), potential mutagenic effects, cellular toxicity, and long-term infection of cells. In contrast, the nucleofection technique is GMP adapted, effective, easy to operate, and thus potentially suitable for an application in patients.^20,21^

In this study, IL-10 was overexpressed in CD11b^+^ monocytes/macrophages by mRNA-nucleofection. These cells were administered in a murine model of autoimmune myocarditis to reduce inflammation and improve clinical outcome. Our therapeutic approach should represent the proof of principle to be transferred to other inflammatory diseases such as rheumatoid arthritis or Crohn disease.

## Methods

### Isolation, Characterization, and Culture of Murine CD11b^+^ Monocytes/Macrophages

A/J mice (Jackson Laboratory, Bar Harbor, ME) >8 weeks old served as donors for macrophage isolation. After isoflurane anesthesia and cervical dislocation, 15 mL of a 10% Hanks' balanced salt solution (HBSS; Gibco, Life Technologies, Grand Island, NY) supplemented with 1% HEPES and 2% FCS, were injected intraperitoneally. After 5 minutes of incubation, the buffer was aspirated with a syringe and the cell suspension was centrifuged at 300*g*. Then the pellet was resuspended in 450 μL of PBS with 0.3% BSA and 2 mmol/L EDTA. Fifty microliters of magnetically labeled anti-mouse/human CD11b MicroBeads (Miltenyi Biotec, Bergisch Gladbach, Germany) was added for 20 minutes. CD11b^+^ monocytes/macrophages were extracted using a mass spectrometry column (Miltenyi Biotec) according to the manufacturer's instructions. Per mouse, ≍0.5 × 10^6^ monocytes/macrophages could be isolated. Monocytes/macrophages of 2 mice were pooled to obtain 1 million cells for subsequent mRNA-nucleofection.

In addition, murine monocytes/macrophages were isolated from the spleen. The spleen was extracted, cut into small pieces, and homogenized by squeezing the cells through a 30-μm preseparation filter (Miltenyi Biotec). Next, CD11b^+^ monocytes/macrophages were purified as described earlier.

### Overexpression of IL-10 in Human Monocytes In Vitro

Human monocytes were isolated from buffy coats purchased from the DRK (German Red Cross) Blood Transfusion Service, Baden-Württemberg-Hessen, Ulm University. In brief, using the lymphocyte separation medium LSM1077 (PAA, Pasching, Austria) and density gradient centrifugation in a Leucosep tube (Greiner Bio-One, Frickenhausen, Germany), we obtained mononuclear cells in the interphase that were removed, washed, and filtered through a 30-μm preseparation filter (Miltenyi Biotec). Then, negative selection of CD14^+^ monocytes was performed through magnetic bead separation according to the manufacturer's instructions (Monocyte Isolation Kit II, Miltenyi Biotec). The purity of CD14^+^ monocytes was >90% as detected by flow cytometry using a phycoerythrin-labeled mouse anti-human CD14 antibody (BD, Heidelberg, Germany; data not shown).

The experimental setting was analog to that of the experiments carried out with murine CD11b^+^ cells, in which 6 × 10^6^ monocytes were nucleofected with 3 or 6 μg of mRNA for human IL-10, respectively. The Human Monocyte Nucleofector Kit (VPA-1007; Lonza, Cologne, Germany) and program Y-01 of the nucleofector device were used. Finally, human IL-10 was measured using a DuoSet ELISA Development System (R&D Systems, Germany). The human IL-10 plasmid (NM_000572, 534 bp) was purchased from Qiagen (Human IL-10 QIAgenes Expression Kit, Qiagen, Hilden, Germany), cloned, and expanded in TOP 10 cells. Linearization was performed with *Sca*I (New England Biolabs, Ipswich, MA).

The experiments were approved by the institutional review committee of Ulm University (Study No. 154/12), and all subjects gave written informed consent.

### mRNA-Nucleofection of Different Monocyte/Macrophage Populations

To establish an optimized protocol for mRNA-nucleofection, 2 different populations of monocytes/macrophages were compared for overexpression of enhanced green fluorescent protein (EGFP) (see earlier): lienal versus peritoneal monocytes/macrophages. Respectively, 3 μg of mRNA was transfected and EGFP expression was measured 24 hours later by fluorescence-activated cell sorter (FACS) analysis. A significantly higher expression of EGFP could be observed in peritoneal than in lienal monocytes/macrophages. On average, we could detect 20% (1% to 40%) EGFP-positive lienal monocytes/macrophages and 82% (80% to 85%) EGFP-positive peritoneal monocytes/macrophages. Three independent experiments were performed. In addition, isolation of monocytes/macrophages from the abdominal cavity was more reproducible compared with the isolation of lienal monocytes/macrophages. As a result of this, peritoneal monocytes/macrophages were isolated in this study (data not shown).

### Preparation of Red Fluorescent Protein–Positive Monocytes/Macrophages

For morphological in vivo tracking of CD11b^+^ administered, monocyte/macrophage cells were isolated from red fluorescent protein (RFP)^+^ transgenic reporter mice. All cells of these mice express RFP, which makes donor monocytes/macrophages visible in the host. RFP^+^-transgenic reporter mice were kindly provided by Prof H. J. Fehling (Institute for Immunology, Ulm University^22^). To avoid cell rejection, we backcrossed the RFP^+^ C57BL/6-inbred reporter mice with A/J mice. To confirm and accelerate the backcross procedure, a genome scanning (Jackson Laboratories) was performed to identify the mice with the most autologous genotype. Thus, a specific backcross to the A/J background was possible.

### Vector Construction and In Vitro Transcription

The murine 537-bp IL-10 gene was cloned into the pBluescript II SK(+) vector and purchased from Eurofins (Ebersberg, Germany). For our experiments, the IL-10 plasmid was diluted 1:10, and the IL-10 gene was cloned into a pcDNA3.1/V5-His TOPO vector (Invitrogen, Carlsbad, CA). The plasmid was enzymatically linearized using *Sca*I, and in vitro transcription was performed as described earlier.^20,21^

### mRNA-Nucleofection of IL-10 and EGFP

Monocytes/macrophages (10^6^) were dissolved in 100 μL of the mouse macrophage Nucleofector solution (AMAXA, Germany), and 3 or 6 μg of IL-10 mRNA was added; for nucleofection, program X-01 of the Nucleofector device was used. Next, monocytes/macrophages were cultured in RPMI medium (PAA) containing 10% FCS and 1% penicillin-streptomycin-glutamine.

Alternatively, 3 μg of mRNA for EGFP was nucleofected. The DNA plasmid that served as a template for in vitro transcription was kindly provided by Dr Peter Ponsaerts (University of Antwerp, Antwerp, Belgium). EGFP-positive cells were detected via FACS using a BD FACS LSR II flow cytometer with BD FACSDiva software (BD Immunocytometry Systems, Heidelberg, Germany) as described in detail.^20,21^

### IL-10 ELISA

#### Time-dependent effects of IL-10 overexpression in vitro

CD11b^+^ macrophages/monocytes (10^6^) were nucleofected with 3 μg of mRNA for IL-10 as described earlier. Cells were seeded onto 48-well plates and cultured for 3, 6, 24, 30, 48, and 72 hours in 500 μL of RPMI (PAA, Pasching, Austria), 10% FCS, and 1% PSG medium per well. The IL-10 concentration was determined in the supernatant using a DuoSet ELISA Development System (R&D Systems). For detection, either a Blue Star HRP Substrate (Diarect AG, Freiburg, Germany) or a TMB One Component HRP Microwell substrate (BioFX SurModics, Eden Prairie, MN) was used. The color reaction was stopped by the addition of 1 mol/L H_2_SO_4_. The ELISA was performed according to the manufacturer's instructions, and detection was performed at a wavelength of 450 nm. In addition, the IL-10 concentration in the supernatant was determined depending on different time intervals after nucleofection (ie, between 0 and 6 hours, 6 and 24 hours, and 24 and 48 hours). The supernatant was removed accordingly to investigate the kinetics of IL-10 de novo production.

#### Dose-dependent effects of IL-10 overexpression in vitro

We used 3 or 6 μg of mRNA for IL-10 for nucleofection. The IL-10 concentration in the supernatant was analyzed 6, 24, and 48 hours later.

#### Determination of IL-10 serum level in vivo

IL-10 levels were determined in the blood serum of healthy WT A/J mice 24 hours after injection of mock-infected and IL-10–nucleofected monocytes/macrophages. No myocarditis was induced in these mice. Then, 10^6^ monocytes/macrophages were transfected, respectively, and 24 hours later IL-10 levels were determined in the serum by ELISA as described earlier.

### Murine Myocarditis Model

#### Experimental animals

Female A/J mice at the age of 6 weeks were purchased from Harlan Laboratories (Harlan Winkelmann GmbH, Borchen, Germany) to induce acute myocarditis as described below.

RFP^+^ transgenic reporter mice were kindly provided by Prof H. J. Fehling.^22^ To avoid cell rejection, we backcrossed the RFP^+^ C57BL/6-inbred reporter mice with A/J mice. To confirm and accelerate the backcross procedure, a genome scanning (Jackson Laboratory) was performed to identify the mice with the most autologous genotype. The number of animals used is indicated at each experiment. All procedures described were in accordance with institutional guidelines.

#### Anesthesia of mice

Donor mice were exposed to 1% (v/v) isoflurane before cervical dislocation followed by monocyte isolation. For echocardiography, mice were investigated after anesthesia with 1% to 2% (v/v) isoflurane (n=10).

#### Production and purification of cardiac troponin I

Murine cardiac troponin I (cTnI) was transformed into *Escherichia coli* bacteria. The protein was expressed and purified by anion-cation exchange and affinity chromatography. The quality of the isolated protein was analyzed by SDS-PAGE and Western blotting. A detailed protocol was given earlier.^23,16,24^

#### Immunization

This procedure was described in detail earlier.^23,16,24^ In brief, for induction of myocardial inflammation, cTnI and complete Freud adjuvant were mixed in a 1:1 ratio. Afterward, an emulsion was generated. Female A/J mice at the age of 6 weeks were subcutaneously injected with 120 μg of the emulsion on days 0 and 7.^23,16^ A control group was immunized with an emulsion of the prefraction and postfraction from the cTnI and complete Freud adjuvant alone.

#### In vivo application of modified monocytes/macrophages

IL-10–overexpressing monocytes/macrophages (4×10^6^) were injected on days 0, 7, and 14 into the tail vein of A/J recipient mice (n=10). To confirm a sufficient mRNA-nucleofection, IL-10 levels were determined in vitro by ELISA in the supernatant of these monocytes/macrophages as described earlier. This represents a prophylactic approach as therapy is initiated simultaneously with triggering of myocardial inflammation. CD11b^+^ cells that underwent the nucleofection procedure without addition of IL-10 mRNA served as controls (ie, mock transfection, n=10).

### Autoantibody Titers Against cTnI

An ELISA was established to measure the titer of autoantibodies against cTnI. The 96-well plates were coated overnight at 48°C with 5 mg/mL cTnI (100 μL/well) dissolved in bicarbonate buffer (NaHCO_3_ 0.1 mol/L, Na_2_CO_3_ 34 mmol/L, pH 9.5). Then, 1× PBS/0.05% Tween 20 served as washing buffer. Plates were then coated with 1% gelatin from cold water fish skin (300 mL/well; Sigma-Aldrich Corp, St Louis, MO). After an incubation period of 2 hours at 37°C and rinsing, IgG (Sigma-Aldrich Corp) diluted to 1:5000 was applied for detection (1 hour at room temperature, 100 mg/well). Dilution series of serum samples were performed as follows: 1:100, 1:400, 1:1600, 1:6400, and 1:25 600. Blue Star HRP substrate solution (Diarect AG) was then applied for 30 minutes at room temperature (100 μL/well) and the color reaction was stopped with 0.3 mol/L H_2_SO_4_. All samples were measured in duplicate. Optical densities of each well were determined using a microplate reader set at 450 nm. The antibody end point titer of each mouse was determined as the highest positive dilution of antibody.

### Functional Analysis in Heart Failure

#### Transthoracic echocardiography

Echocardiography was performed in the standard views using an ATL-HDI 9000 device (Philips, the Netherlands) with a 10-MHz linear transducer. In the longitudinal axis, end-diastolic and end-systolic diameters, ejection fraction, fractional shortening, and heart rate were determined.

#### Treadmill exercise test

A running wheel was placed in each cage and every mouse had its own device. The individual voluntary walking distance and time were measured. After 1 week of adaption the parameters obtained in the third week after immunization with cTnI were analyzed.

### Histological Analysis

#### Determination of myocardial inflammation and fibrosis

Serial cross sections of 5-μm thickness, each through the entire heart, were prepared and stained with hematoxylin/eosin to determine the level of inflammation, or stained with Masson's trichrome to define the extent of collagen deposition/fibrosis. Two independent examiners blinded to the treatment arm of the respective specimens explored every fifth cross section, and histoscores and fibrosis scores were assigned according to the 6-tier scoring system published previously (grade 0, no inflammation; grade 1, cardiac infiltration in up to 5% of the cardiac sections; grade 2, infiltration in 6% to 10%; grade 3, infiltration in 11% to 30%; grade 4, infiltration in 31% to 50%; and grade 5, infiltration in 50% of cardiac sections). The fibrosis score was determined analogous to the histoscore.^23,16,24^

#### Determination of myocardial fibrosis

Masson's trichrome staining was used to determine the extent of collagen deposition. Myocardial fibrosis was semiquantitatively analyzed according to the pattern above.^23,16,24^

#### Immunohistochemical analysis of myocardial inflammation

Myocardial inflammation was assessed by immunohistochemical analysis as described earlier.^25^ In brief, CD68^+^ macrophages and IL-10 were detected within the myocardium with use of the avidin-biotin-peroxidase complex. Myocardial sections were fixed with formalin and incubated with 0.1% proteinase (Sigma-Aldrich Corp) for 10 minutes. A 1% H_2_O_2_/methanol solution was used to block endogenous peroxidase activity. Unspecific antigens were blocked with donkey or goat serum in a 1:10 dilution. A polyclonal rat anti-mouse CD68 antibody (dilution 1:50; clone FA-11, Acris Antibody GmbH, Herford, Germany) and a polyclonal goat anti-mouse IL-10 M-18 antibody (dilution 1:50; Santa Cruz Biotechnology, Santa Cruz, CA) were used as a primary antibody. A biotinylated goat anti-rat antibody (CD68; dilution 1:200; BioLegend, San Diego, CA) and donkey anti-goat antibody (IL-10; dilution 1:500; Dianova, Hamburg, Germany) served as a secondary antibody, respectively. Myocardial sections were analyzed every 25 μm for each antigen using an Axioskop 2 plus microscope (Zeiss, Germany). Thus, per mouse at least 20 sections were available, which represent ≍400 high-power fields (ie, ×40 magnification). A semiquantitative score system was applied for IL-10. Staining intensity was indicated from “0” (ie, no IL-10 detection) to “+++” (ie, strong IL-10 detection). CD68^+^ cells were counted under the light microscope and presented as cells/mm^2^. Immunohistochemical analysis was performed in a blinded manner by experienced investigators.

### Flow Cytometry Analysis

Mice were euthanized at indicated times by CO_2_ inhalation, and spleens and lymph nodes were isolated. Tissues were macerated on cell strainers (BD Biosciences) in HBSS (Life Technologies) supplemented with 1% HEPES and 2% FBS. Cell suspensions were centrifuged at 350*g* and 8°C, washed once in PBS (BSA 0.5%, EDTA 2 mmol/L), and incubated in red blood cell lysis buffer (NH_4_Cl 155 mmol/L, NaHCO_3_12 mmol/L, EDTA 0.1 mmol/L, pH 7.2) for 5 minutes at room temperature. After 2 washes in PBS, 10^6^ cells per well were seeded onto a 96-well plate, and nonspecific binding was blocked by incubation with rat anti-mouse CD16/CD32 antibody (dilution 1:10; BD PharMingen, Heidelberg, Germany) for 15 minutes at 4°C. Then, cells were washed in PBS, stained with fluorescein isothiocyanate anti-mouse CD11b (BD Pharmingen) for 1 hour at 4°C, and washed twice in PBS, and fluorescence intensities were acquired on an FACSAria cell sorter (BD Biosceinces).

### Statistical Analysis

Data were analyzed using Kruskal–Wallis test followed by Mann–Whitney *U* test to explore the significance between treatment groups. A value of *P*<0.05 was considered significant. SPSS (version 15.0; SPSS Inc, Chicago, IL) statistical software was used for all calculations.

## Results

### Murine IL-10 ELISA

#### Time-dependent effects in vitro

**Figure 1.**
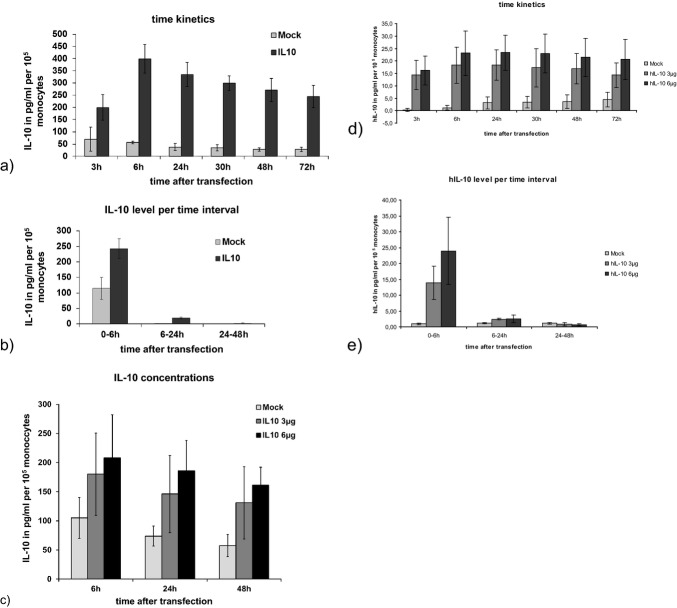
a–c, IL-10 expression in murine monocytes/macrophages in vitro. a, Mean IL-10 levels were analyzed after nucleofection of IL-10 and mock-transfected mRNA (n=3, respectively). b, The mean IL-10 production was analyzed for different time intervals depending on the time point after nucleofection (n=3). c, The mean IL-10 concentration was analyzed after nucleofection of 3 or 6 μg mRNA (n=4). d and e, IL-10 expression in human monocytes in vitro. d, Mean IL-10 levels were analyzed after nucleofection of 3 and 6 μg of mRNA for IL-10 and compared with mock control (n=4). e, The mean IL-10 production was analyzed for different time intervals depending on the time point after nucleofection (n=3).

Six hours after mRNA-nucleofection, the maximum IL-10 concentration was detected. For each 10^5^ monocytes/macrophages, the IL-10 concentration was 399±59 pg/mL in the positive group and 57±6 pg/mL in the control group. Because mRNA-nucleofection represents a transient form of cell modification, IL-10 levels constantly decreased afterward (Figure 1a). Nearly all IL-10 was produced within the first 6 hours after mRNA-transfection (Figure 1b).

#### Dose-dependent effects in vitro

A significant increase in IL-10 production was observed when nucleofection was performed with 6 μg of mRNA instead of with 3 μg of mRNA (Figure 1c).

### Human IL-10 ELISA

Basically, the earlier described time- and dose-dependent effects of IL-10 overexpression in mice could be reproduced in human monocytes in vitro (Figure 1d and 1e).

### Targeted Therapy

#### Morphological tracking of monocytes/macrophages by RFP

**Figure 2.**
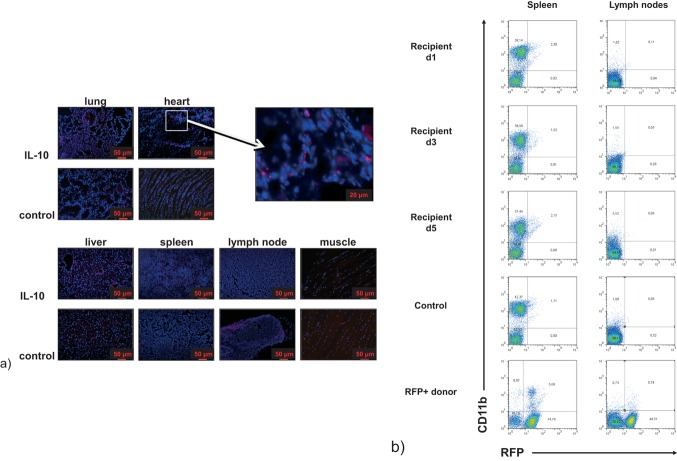
Histological analysis. a, Morphological tracking of monocytes/macrophages by RFP. No RFP^+^ monocytes/macrophages could be detected within the lung, liver, muscle, kidney, and skin (data not shown). In the spleen and lymph nodes, a weak but unspecific background staining could be seen. Significantly more RFP^+^ monocytes/macrophages could be detected within the heart of IL-10–treated mice compared with the control (representative photographs). b, FACS analysis of different organs for RFP and CD11b. Cell suspensions derived from spleens and lymph nodes of mice euthanized 1, 3, or 5 days after IL-10–nucleofected monocyte/macrophage transfer, mock-nucleofected monocyte/macrophage–recipient mice (control), or donor mice were analyzed by flow cytometry for RFP and CD11b expression. Indicated numbers represent the cell percentages of the respective cell subpopulations: CD11b^+^RFP^−^ (*top left*); CD11b^+^RFP^+^ (*top right*); CD11b^−^RFP^−^ (*bottom left*); CD11b^−^RFP^+^ (*bottom right*). Fluorescence intensities of at least 30 000 events per sample were acquired. RFP indicates red fluorescent protein; FACS, fluorescence-activated cell sorter.

For morphological tracking of macrophage migration into the inflammatory tissue, myocarditis was induced in RFP^−^ A/J mice. IL-10 was overexpressed in monocytes/macrophages isolated from RFP^+^ mice and was administered on day 20 into the tail vein of immunized RFP^−^ mice. A healthy A/J wild-type mouse served as a control. Twenty-one days after immunization, the recipient mice were euthanized, and the heart, lung, kidney, liver, spleen, skin, muscle, and lymph nodes were analyzed for RFP^+^ and CD11b^+^ cells. No significant number of RFP^+^ monocytes/macrophages could be detected within the lungs, liver, muscle (Figure 2a), kidney, and skin (data not shown). In the spleen and lymph nodes, a weak red background signal could be detected. In relation to the 4′,6-diamidino-2-phenylindole–stained cell nuclei, this signal was unspecific and no differences were seen compared with the control mouse. Similarly, quantitative flow cytometric analysis did not detect RFP^+^ monocytes/macrophages in these tissues (Figure 2b). In the study group, a significant number of RFP^+^ monocytes/macrophages could be detected within the myocardium (inset, Figure 2a). This finding indicates a directed migration of RFP^+^ monocytes/macrophages to sites of inflammation and opens a new field of inflammation-targeted therapy.

**Figure 3.**
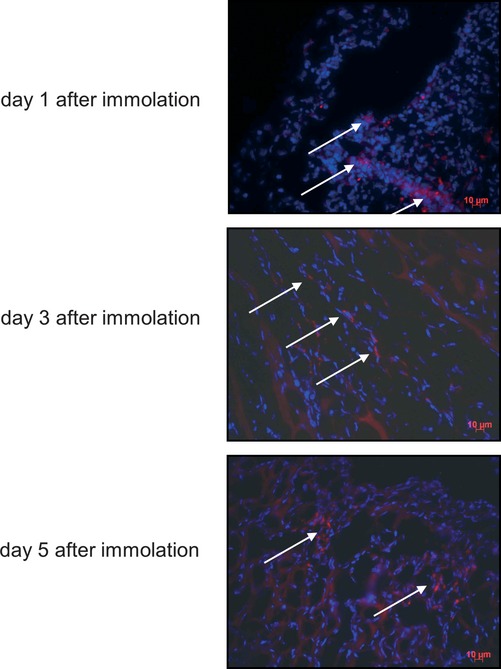
Immunofluorescence microscopy of myocardium for RFP^+^ macrophages. Recipient mice were euthanized 1, 3, and 5 days after IL-10–nucleofected RFP^+^ donor macrophages were administered. The myocardium of the recipient mice was investigated by immunofluorescence microscopy for RFP^+^ macrophages. All time points indicated RFP^+^ donor macrophages could be detected within the myocardium of the recipient mice (*white arrows*). RFP indicates red fluorescent protein.

RFP^+^ macrophages could be detected within the myocardium of the recipient mice 1, 3, and 5 days after intravenous application (Figure 3).

#### Functional tracking of monocytes/macrophages by IL-10 production

**Figure 4.**
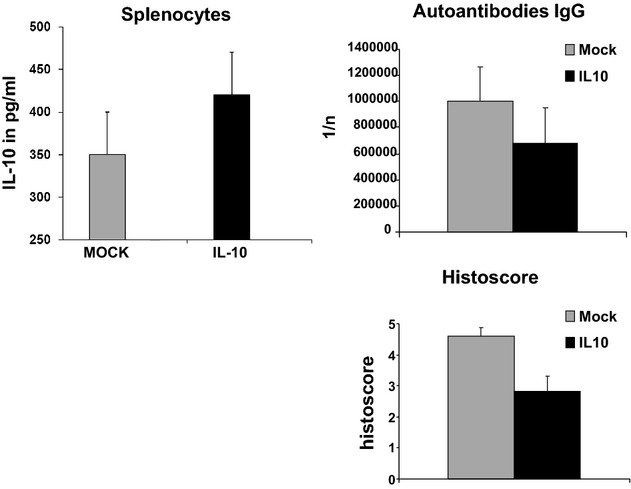
Functional tracking of monocytes/macrophages by IL-10: In splenocytes of wild-type A/J mice, a basal IL-10 production was observed after administration of mock-transfected monocytes/macrophages. Significantly more IL-10 was detected after IL-10–overexpressing monocytes/macrophages were intravenously injected. Anti-cTnI antibody titers: Mice treated with IL-10–overexpressing monocytes/macrophages showed significantly lower titers of anti-cTnI autoantibodies compared with mock-transfected mice. Histoscore: Myocardial inflammation was significantly decreased after treatment with IL-10–overexpressing monocytes/macrophages compared with controls.

IL-10–overexpressing CD11b^+^ cells were injected intravenously into healthy wild-type A/J mice without induction of myocarditis. Twenty-four hours later, IL-10 level was determined in the splenocytes. The mean IL-10 level was 420 pg/mL in the treatment group versus 350 pg/mL in the mock-infected control group (*P*<0.05; Figure 4a). This result indicates that in healthy animals without active inflammation, IL-10–overexpressing monocytes/macrophages leave the circulation to be stored within the spleen.

**Figure 5.**
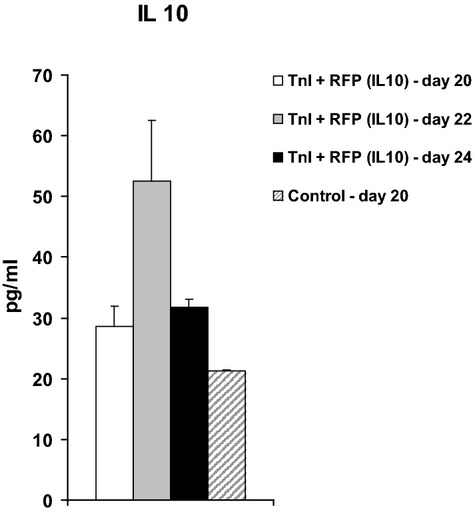
IL-10 ELISA of dissociated heart tissue. On days 1, 3, and 5 after the last intravenous injection of RFP^+^ macrophages, the hearts of the recipient mice were dissociated. An ELISA was performed for myocardial IL-10. One mouse that was not treated with IL-10–overexpressing macrophages was euthanized on day 1 and served as a control. This mouse indicated the basal expression of IL-10 within the myocardium. RFP indicates red fluorescent protein; ELISA, enzyme linked immunosorbent assay.

On days 1, 3, and 5 after the last intravenous injection of RFP^+^ macrophages, significant amounts of IL-10 could be detected within the myocardium of the recipient mice with ELISA. The highest IL-10 concentration was observed on day 3. At all time points, IL-10 concentrations were above the baseline level determined in a control mouse (Figure 5).

#### Determination of IL-10 serum level in vivo

Twenty-four hours after injection of mock- and IL-10–nucleofected CD11b^+^ cells, IL-10 levels were determined in the serum of healthy WT A/J mice. Myocarditis was not induced in these mice. In both groups, the IL-10 level were below the minimum detection limit of 4.0 pg/mL (data not shown). Because no relevant serum IL-10 levels were observed for both groups, adverse systemic effects of this therapy might be missing.

### Histological Analysis

#### Histoscore

A significant reduction in infiltrating cells could be seen within the myocardium after the application of IL-10–overexpressing monocytes/macrophages (Figure 4c). The histoscore was 2.9±0.6 for IL-10–treated mice versus 4.6±0.4 for the control mice (*P*<0.01).

#### Immunohistochemical analysis

**Figure 6.**
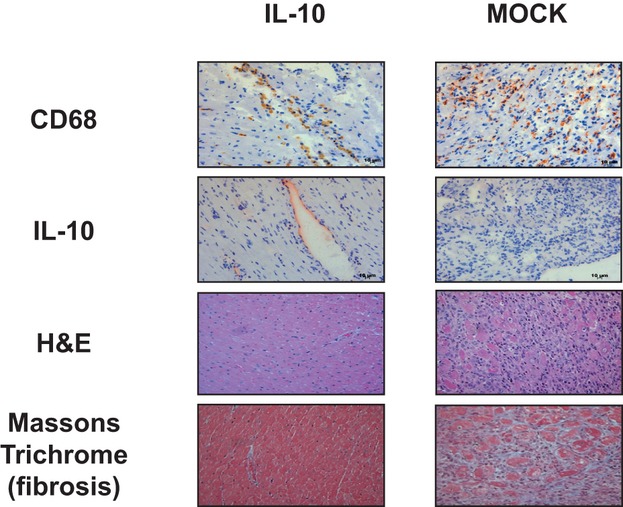
Immunohistochemical staining. By trend, more IL-10, less macrophages (CD68), less infiltrating cells (hematoxylin -eosin), and less fibrosis were detected in IL-10–treated mice (n=4, respectively). MOCK indicates mock-transfected cells.

**Figure 7.**
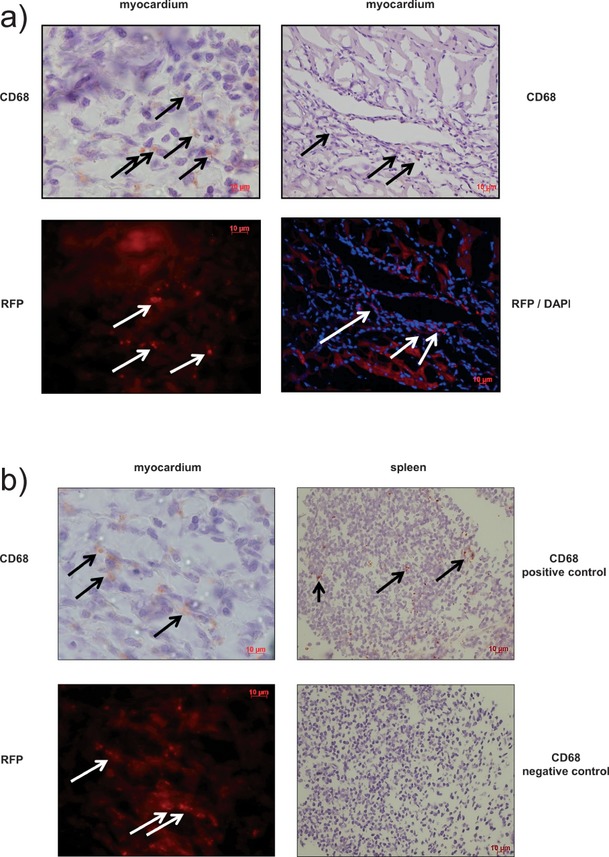
Colocalization of RFP with macrophages (CD68). a and b, In sequential sections, immunohistochemical staining for CD68 (*brown*) identifies RFP-positive cells (*red*) as macrophages (*left*). Previously these cells have been injected intravenously. 4′,6-Diamidino-2-phenylindole (DAPI) staining (*blue*) indicates the cell nuclei. Different magnifications are shown. Not all macrophages (*brown*) colocalize with the red cells as RFP is specific for intravenously injected donor macrophages. Consequently, macrophages (*brown*) showing no RFP colocalization represent host macrophages. b, Right: Sections of the spleen served as a positive control for macrophage staining. Omitting the secondary antibody represented the negative control. RFP indicates red fluorescent protein.

We observed less infiltration of CD11b^+^ cells and more expression of IL-10 in the myocardium of IL-10–treated mice compared with the control mice. In IL-10–treated mice, the macrophage count was 35.8 macrophages/mm^2^ and relevant amounts of IL-10 were detected (ie, “++”). In the control mice, the macrophage count was 63.7 macrophages/mm^2^ and almost no IL-10 (ie, “+”) was seen (Figure 6). RFP^+^ macrophages colocalized with the CD68 antigen (Figure 7a and 7b).

#### Myocardial fibrosis

There also was a significant reduction in fibrosis. The fibrosis score was 2.6±0.5 for IL-10–treated mice versus 4.3±0.4 for the control mice (*P*<0.01; Figure 6).

### Autoantibody Titer

Mice treated with IL-10–overexpressing CD11b^+^ cells showed significantly lower titers of anti-cTnI autoantibodies (676 073±279 033) compared with mock-treated animals (1 003 520±259 862; *P*<0.05; Figure 4b).

### Functional Analysis

#### Echocardiography

**Figure 8.**
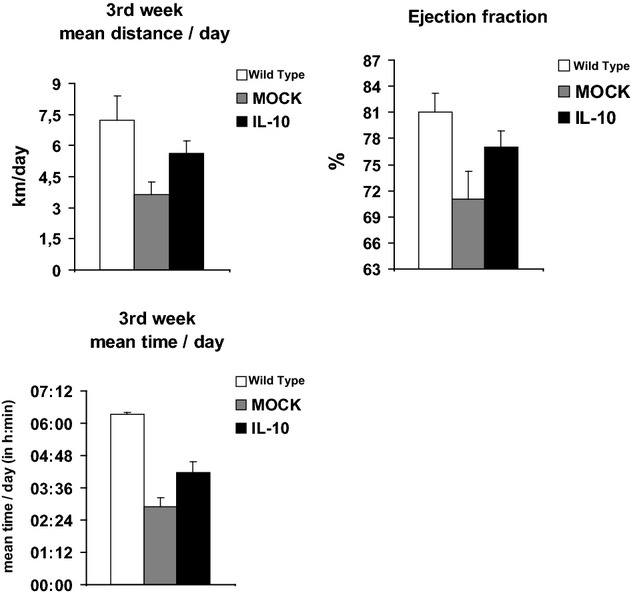
Functional analysis. a, Physical performance was increased in IL-10–treated mice as mean walking distance per day (*top*, *P*=0.02) and mean exercise time per day (*bottom*, *P*=0.04) increased significantly. Healthy A/J wild-type mice represent the controls. b, By trend, a better left ventricular ejection fraction was detected for IL-10–treated mice (*P*=0.16). Healthy A/J wild-type mice represent the controls. MOCK indicates mock-transfected cells.

By trend, IL-10 treatment resulted in a slightly better ejection fraction (77.0±2.0%) than did application of mock-transfected monocytes/macrophages (71.0±3.0%). This increase was small and statistically nonsignificant (*P*=0.16; Figure 8b).

#### Treadmill test

The mean walking distance per day was 5.6±0.7 km in the treatment group versus 3.8±0.6 km in the control group (*P*=0.02). The mean exercise time per day was increased compared with mock-transfected controls (4 hours12 minutes±28 minutes versus 2 hours 54 minutes±20 minutes; *P*=0.04; Figure 8a).

### Dose-Dependent Effects of IL-10 Therapy

The described experiments were reproduced using 1×10^6^ instead of 4×10^6^ IL-10–overexpressing CD11b^+^ cells. We were unable to detect any significant differences for functional tests and histological analysis (not shown).

## Discussion

In this study, we investigated an in vivo application of IL-10–overexpressing monocytes/macrophages for anti-inflammatory therapy in a murine model of autoimmune myocarditis. We demonstrated that myocardial inflammation could be reduced and exercise performance was improved after injection of genetically modified monocytes/macrophages. We gained evidence that our therapeutic approach displayed a local action that was focused within the myocardium as we could not detect an elevated IL-10 plasma level after intravenous injection of IL-10–overexpressing monocytes/macrophages. Significantly more RFP^+^ monocytes/macrophages could be detected within the myocardium of cTnI-immunized mice in contrast to other tissues. We postulate that monocytes/macrophages rather selectively migrate into inflamed tissues, release IL-10 locally, and thus mediate their beneficial anti-inflammatory effects specifically.

Within recent years, there were numerous attempts to extend standard heart failure therapy in myocarditis. Unspecific immunosuppressive therapy using prednisone, cyclosporine, or azathioprine was introduced without convincing results.^8^ In viral dilated cardiomyopathy, a specific antiviral therapy with interferon β1b was investigated. In an initial phase II study, some beneficial effects could be detected for this therapy,^11^ but a second phase II study and an independent clinical observation could not confirm the first promising results.^26,27^ Against this background, new anti-inflammatory therapies based on IL-10 overexpression seem worthwhile to follow.

The anti-inflammatory power of the Th2-associated cytokine IL-10 has been used in several recent studies. IL-10 mediates its immunomodulatory properties via inhibition of Th1 cells, monocytes/macrophages, and cytokines such as nuclear factor-κB, tumor necrosis factor-α, IL-1, or IL-6 and thus protects the organism from overwhelming proinflammatory conditions.^28^ Recently, IL-10 was suggested as the effective part of some anti-inflammatory therapies as fenofibrate,^29^ quercetin (a flavonoid),^30^ mesenchymal stem cells,^31^ immunoglobulins,^32^ and methotrexate.^33^ Nishio and colleagues administered recombinant IL-10 subcutaneously in a murine model of autoimmune myocarditis caused by the encephalomyocarditis virus.^18^ They could report a significantly higher survival rate in the treatment group compared with the control group. Furthermore, myocardial lesions were smaller and the levels of tumor necrosis factor-α, IL-2 and inducible nitric oxide synthase within the heart were lower in IL-10–treated mice. Interestingly, the beneficial effects were only seen when IL-10 was begun on the day of virus inoculation (ie, prophylaxis), whereas no effects were seen when IL-10 was administered later (ie, therapy).^18^ This observation could be confirmed in a murine model of fatal group B *Streptococcus* sepsis. In a rat model of myocarditis, IL-10 was also protective when a plasmid vector expressing the IL-10 cDNA was transferred into the tibialis anterior muscle via electroporation.^34,35^ Recently, we demonstrated the potential of adeno-associated virus (AAV)-9–mediated cardiac expression of IL-10 for the prevention of autoimmune myocarditis.^36^ These treated mice showed not only increased IL-10 expression in the myocardium but also high serum IL-10 levels. Despite the high tropism for adult cardiomyocytes of AAV-9, some hepatic AAV-9 infection could be found.^36^

A clinical in vivo transfer of gene therapy is still challenging. Mostly viral or AAV strategies are used. The transfer of these therapy approaches into clinical setups is mostly limited by systemic side effects, potential mutagenic effects, potential cellular and/or organ toxicity, long-term infection of cells, and potential procedural toxicity.^17,19,37^ In this study, we report for the first time that IL-10 can efficiently be overexpressed in monocytes/macrophages by mRNA-nucleofection. Furthermore, we demonstrated that the application of these IL-10–overexpressing monocytes/macrophages decreased inflammation and improved physical performance in a murine model of autoimmune myocarditis. Our therapeutic approach is adapted to the GMP guidelines,^20,21^ and isolation of the monocytes can be arranged easily in an autologous setting. Furthermore, successful IL-10 overexpression could also been demonstrated in human monocytes in vitro (Figure 1d and 1e). Thus, basically a bridge to a clinical application in patients can be built. Because nucleofection results in a transient overexpression of IL-10, there is no permanent genetic modification of the injected monocytes/macrophages, which further supports their clinical use.^20,21^ Modifications of the mRNA or use of an IL-10 DNA plasmid for nucleofection could potentially influence the duration of IL-10 expression.

Finally, we present the first proof of principle for a new therapeutic approach in autoimmune myocarditis. As this therapy represents an universal approach for a targeted anti-inflammatory therapy it could be transferred to other processes characterized by local inflammation such as Crohn disease, vasculitis, rheumatoid arthritis, and others.
